# Differential insulin sensitivity of NMR-based metabolomic measures in a two-step hyperinsulinemic euglycemic clamp study

**DOI:** 10.1007/s11306-021-01806-2

**Published:** 2021-06-09

**Authors:** Wenyi Wang, Ko Willems van Dijk, Carolien A. Wijsman, Maarten P. Rozing, Simon P. Mooijaart, Marian Beekman, P. Eline Slagboom, J. Wouter Jukema, Raymond Noordam, Diana van Heemst

**Affiliations:** 1 Department of Human Genetics, Leiden University Medical Center, Leiden, The Netherlands; 2 Division of Endocrinology, Department of Internal Medicine, Leiden University Medical Center, Leiden, The Netherlands; 3 Section of Gerontology and Geriatrics; Department of Internal Medicine, Leiden University Medical Center, PO Box 9600, 2300RC Leiden, The Netherlands; 4 Department of Public Health and Institute of Clinical Medicine, Psychiatric Centre Copenhagen, University of Copenhagen, Copenhagen, Denmark; 5 Section of Epidemiology, University of Copenhagen, Copenhagen, Denmark; 6 The Research Unit for General Practice and Section of General Practice, University of Copenhagen, Copenhagen, Denmark; 7 Section of Molecular Epidemiology, Department of Biomedical Data Sciences, Leiden University Medical Center, Leiden, The Netherlands; 8 Max Planck Institute for Biology on Ageing, Cologne, Germany; 9 Department of Cardiology, Leiden University Medical Center, Leiden, The Netherlands; 10 Netherlands Heart Institute, Utrecht, The Netherlands

**Keywords:** Insulin resistance, Hyperinsulinemic euglycemic clamp study, Metabolomic measures

## Abstract

**Background:**

Insulin is the key regulator of glucose metabolism, but it is difficult to dissect direct insulin from glucose-induced effects. We aimed to investigate the effects of hyperinsulemia on metabolomic measures under euglycemic conditions in nondiabetic participants.

**Methods:**

We assessed concentrations of 151 metabolomic measures throughout a two-step hyperinsulinemic euglycemic clamp procedure. We included 24 participants (50% women, mean age = 62 [s.d. = 4.2] years) and metabolomic measures were assessed under baseline, low-dose (10 mU/m^2^/min) and high-dose (40 mU/m^2^/min) insulin conditions. The effects of low- and high-dose insulin infusion on metabolomic measures were analyzed using linear mixed-effect models for repeated measures.

**Results:**

After low-dose insulin infusion, 90 metabolomic measures changed in concentration (p < 1.34e^−4^), among which glycerol (beta [Confidence Interval] =  − 1.41 [− 1.54, − 1.27] s.d., p = 1.28e^−95^) and three-hydroxybutyrate (− 1.22 [− 1.36, − 1.07] s.d., p = 1.44e^−61^) showed largest effect sizes. After high-dose insulin infusion, 121 metabolomic measures changed in concentration, among which branched-chain amino acids showed the largest additional decrease compared with low-dose insulin infusion (e.g., Leucine, − 1.78 [− 1.88, − 1.69] s.d., P = 2.7e^−295^). More specifically, after low- and high-dose insulin infusion, the distribution of the lipoproteins shifted towards more LDL-sized particles with decreased mean diameters.

**Conclusion:**

Metabolomic measures are differentially insulin sensitive and may thus be differentially affected by the development of insulin resistance. Moreover, our data suggests insulin directly affects metabolomic measures previously associated with increased cardiovascular disease risk.

**Supplementary Information:**

The online version contains supplementary material available at 10.1007/s11306-021-01806-2.

## Introduction

Insulin is an anabolic peptide hormone secreted by the pancreas in response to increased blood glucose levels to activate various mechanisms that decrease blood glucose levels (Tokarz et al., 2018). Insulin has broad metabolic effects, which include increasing the rate of glycolysis in fat and muscle, decreasing the rate of lipolysis in fat, decreasing the rate of fatty acid oxidation in muscle and liver, and increasing the rate of protein synthesis in fat, muscle and liver tissue (Dimitriadis et al., 2011; Phillips, 2008). Insulin resistance is a common pathophysiological consequence of obesity in which body cells are unable to raise a potent physiological response to insulin. Insulin resistance precedes the development of type 2 diabetes and is an independent risk factor of cardiovascular disease (Ormazabal et al., 2018; Roberts et al., 2013; Taylor, 2012).

Insulin sensitivity is frequently assessed on the basis of the ratio between fasting insulin and glucose levels calculated as the homeostatic model assessment for insulin resistance (HOMA-IR) index (Gutch et al., 2015). However, an abnormal HOMA-IR index does not provide insight into the tissue-specific origin of the insulin resistance. Insulin not only increases glucose uptake by peripheral tissues such as muscle and fat, but insulin also decreases endogenous glucose production through suppression of gluconeogenesis in the liver and both processes may be affected differentially by insulin resistance (Wallace et al., 2004). A two-step hyperinsulinemic euglycemic clamp analysis was used to assess whole-body insulin sensitivity and a glucose tracer was included to distinguish hepatic and peripheral insulin resistance (Finegood et al., 1987; Muniyappa et al., 2008; Steele, 1959). During the first step a low dose insulin will predominantly act on the liver, whereas during the second step the higher dose will also have a major effect on peripheral tissues such as muscle and fat tissue (Saccà et al., 1982). Administration of a low insulin dose has thus been used to assess the insulin sensitivity of endogenous glucose production by the liver, while administration of a higher insulin dose has been used to additionally assess the insulin sensitivity of glucose uptake by peripheral tissues, particularly skeletal muscle and fat (Bazotte et al., 2014).

Metabolomic measures are thought to reflect the interaction between proteins encoded by the genome and the environment, such as diet and lifestyle (Beger, 2016). Numerous platforms have become available which can be exploited to determine the concentrations of a plethora of metabolomic measures in cells and body fluids (Bukowiecka-Matusiak et al., 2016; Liu & Locasale, 2017). Metabolomic measures have been performed to characterize the response to glucose administration in individuals with varying levels of insulin sensitivity (Shaham, 2008; Wang, 2019). These analyses have provided insight into the physiological responses and pathophysiological processes underlying disease (Wishart, 2019).

Previous studies have shown that multiple blood metabolomic measures are associated with increased insulin resistance and type 2 diabetes (Knebel, 2016; Yang et al., 2018). However, the specific effects of hyperinsulinemia, in the absence of major changes in blood glucose levels, on liver and peripheral tissues in determining blood metabolomic measures have not been fully described in healthy individuals. Therefore, the aim of this study was to investigate the responses of metabolomic measures to two different insulin dosages in a two-step hyperinsulinemic euglycemic clamp study in healthy middle-aged individuals without diabetes mellitus.

## Methods

### Study population and study design

All participants were selected from the Leiden Longevity Study (LLS) (Schoenmaker, 2006). Participants were selected based on the following inclusion criteria: middle-age (50–75 years old), BMI from 22 to 30 kg/m^2^ and living in the proximity of the research center (< 45 min by car). Exclusion criteria were: (1) fasting plasma glucose > 6.9 mmol/L (American Diabetes, 2010); (2) presence of endocrine, renal, hepatic or other significant chronic diseases; (3) use of medication known to influence lipolysis, glucose metabolism or growth hormone secretion; (4) recent weight changes or attempts to lose weight (> 3 kg weight change within last 3 months); (5) smoking; (6) extensive sporting activities (> 10 h per week); (7) inaccessible peripheral veins for intravenous catheter insertion for the assessment by clinical examination and routine laboratory tests. Of the 87 participants that were approached, 17 participants did not fulfill the inclusion criteria (19%), 44 participants refused participation (51%), and 26 participants agreed to participate in the study (30%). Two participants did not finish the study due to medical technical reasons. In total, 24 participants were included in this experiment. Sixteen individuals participated as couples (eight couples) and eight participated as singletons. The Medical Ethical Committee of the Leiden University Medical Center (LUMC) approved the design of the study and all participants gave their written informed consent.

**Fig. 1:**
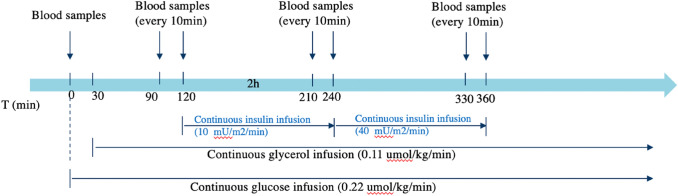
Study design of the two-step hyperinsulinemic euglycemic study

Serum samples were acquired during a two-step hyperinsulinemic euglycemic clamp study (Fig. 1). All clamp studies started at 8:00 in the morning after an overnight fast. At 08:30 h (t = 0 min), an adjusted primed (17.6 μmol/kg) continuous infusion (0.22 μmol/kg/min) of [6,6-^2^H2] glucose (enrichment 99.9%; Cambridge Isotopes, Cambridge, MA, USA) was started and lasted for 360 min. At 9:00 h (t = 30 min), a primed (1.6 μmol/kg), continuous (0.11 μmol/kg/min) infusion of [^2^H_5_]-glycerol (Cambridge Isotopes) was started and continued throughout the study. After two hours of glucose infusion (t = 120 min), low dose human recombinant insulin (10 mU/m^2^/min, Actrapid, Novo Nordisk Pharma BV, Alphen aan den Rijn, the Netherlands) was infused continuously for 2 h. After this, high dose insulin (40 mU/m^2^/min) was infused (t = 240 min) for 2 h. During the insulin infusion, exogenous glucose 20% enriched with 3% [6,6‐^2^H_2_] ‐glucose was infused at a variable rate to maintain the plasma glucose level at approximately 5.0 mmol/L. Blood samples were taken at the start of the study, and subsequently every 10 min from 90 to 120, from 210 to 240 and from 330 to 360 min. All participants underwent a two-step hyperinsulinemic euglycemic protocol and blood samples were taken for the measurement of 151 metabolomic measures (Fig. 1). For the three examined conditions, we measured 3 samples as the baseline sample (measured at 95, 105 and 115 min after the start), 4 samples as low-dose insulin (measured at 210, 220, 230 and 240 min after the start), and another 4 samples as high-dose insulin (measured at 330, 340, 350, 360 min after the start). The study population and study design have been described in more detail elsewhere (Wijsman, 2011).

### Metabolomics analysis

151 lipid and metabolite concentrations in fasting serum samples (ratios not included) were measured using a high-throughput proton NMR metabolomics platform (Nightingale Health Ltd., Helsinki, Finland) (Soininen et al., 2015). This method provides quantification of lipoprotein subclass profiling with lipid concentrations within 14 lipoprotein subclasses. The 14 subclass sizes were defined as follows: extremely large VLDL with particle diameters from 75 nm upwards and a possible contribution of chylomicrons, five VLDL subclasses (average particle diameters of 64.0 nm, 53.6 nm, 44.5 nm, 36.8 nm, and 31.3 nm), IDL (28.6 nm), three LDL subclasses (25.5 nm, 23.0 nm, and 18.7 nm), and four HDL subclasses (14.3 nm, 12.1 nm, 10.9 nm, and 8.7 nm). Within the lipoprotein subclasses the following components were quantified: total cholesterol, total lipids, phospholipids, free cholesterol, cholesteryl esters, and triglycerides. The mean size for VLDL, LDL and HDL particles were calculated by weighting the corresponding subclass diameters with their particle concentrations. Furthermore, the majority of the metabolomic measures that were determined belong to classes of apolipoproteins, cholesterol, fatty acids, glycerides, phospholipids, amino acids, fluid balance, glycolysis-related metabolites, inflammation, and ketone bodies. Detailed experimentation and applications of the NMR metabolomics platform have been described previously (Soininen et al., 2015), as well as representative coefficients of variations (CVs) for the metabolomic measures (Kettunen, 2016).

### Statistical analyses

Characteristics of the study population were presented as percentages (for dichotomous variables) and mean values (with standard deviation [s.d.]).

Missing metabolomic measurement data, which was most frequently due to levels below the limit of detection, were imputed by the half of the minimum of the measured value in the dataset for a specific metabolomic measure. During visual inspection of the data, we observed a decrease in the concentration of albumin during the course of the experiment (Supplementary Fig. 1), which indicates that blood concentrations were increasingly diluted over time of the study period, as found previously (Li & Ji, 2005b). Therefore, with the exception of the VLDL diameter, LDL diameter, HDL diameter, estimated description of fatty acid chain length, and estimated degree of unsaturation (being all not expressed as mmol/L), correction for dilution of metabolomic measure concentrations was done by normalization to the concentration of albumin. This was done by dividing the concentrations of metabolomic measures by the concentration of albumin (consequently concentrations are expressed per mmol/L/mmol/L albumin). After this correction step, data was log-transformed and subsequently standardized (mean = 0, s.d. = 1) to approximate a normal distribution and to make all metabolomic measures comparable in unit and in magnitude of effect. Outliers were defined as a value with > 4 s.d. from the mean, and were excluded from the dataset for the analyses prior to any further analyses. Taking into account time-dependent within-person variation in concentrations of the metabolomic measures, a linear mixed-effect model for repeated measures was applied to explore the changes in metabolomic measures’ concentrations dependent on different insulin infusion doses within individuals (including the difference between two dose groups compared with the baseline measurement and differences between low dose group and high dose group). In order to further explore the insulin sensitivity of branched chain amino acids (BCAA), we calculated the percentage changes of the BCAAs after high dose insulin infusion compared with baseline for all individuals. Subsequently, we assessed the correlation of between the percentage change in BCAAs with glucose infusion rate (GIR), which is a measure of whole-body insulin sensitivity, and with glucose disposal rate (GDR), which is a measure of peripheral insulin sensitivity.

The statistical analyses were conducted in the R software (Version 3.6.2), and subsequent data visualization was performed in either Python (2.7) or using the ggplot2 package in R (R Development Core Team, 2019).

We corrected the results for multiple testing using Bonferroni. As conventional Bonferroni correction is too stringent given the high correlations between multiple of the included metabolic measures, we corrected for the number of independent metabolic measures instead, using methodology that has been described before by Li and Ji (2005a). Based on this method, we corrected for 37 independent metabolomic measures. Hence, we considered a P-value of 0.00134 (notably 0.05/37) the threshold for statistical significance.

## Results

### Characteristics of the study population and metabolomic measures

The characteristics of all participants are shown in Table 1. In total, 24 participants comprising 12 women and 12 men were included in this study. These participants were clinically healthy with a mean age of 62 (s.d. = 4.2) years, mean body mass index of 25.8 kg/m^2^ (s.d. = 1.8), mean fasting plasma glucose of 5.0 mmol/L (s.d. = 0.5) and mean fasting plasma insulin of 6.2 mU/L (s.d. = 2.8). Average values of each metabolomic measure within different dose groups were provided in Supplementary Table 1.

**Table 1:** Characteristics of study population

Characteristics	Total
N	24
Men, N (%)	12 (50)
Age in years, mean (s.d.)	62.0 (4.2)
Body mass index in kg/m^2^, mean (s.d.)	25.8 (1.8)
Plasma glucose after fasting in mmol/L, mean (s.d.)*	5.0 (0.5)
Plasma insulin after fasting in mU/L, mean (s.d.)*	6.2 (2.8)

*Plasma glucose and plasma insulin after fasting were calculated based on 17 participants due to the missing values of 7 participants

### Changes in metabolomic measures at low-dose insulin infusion

**Fig. 2:**
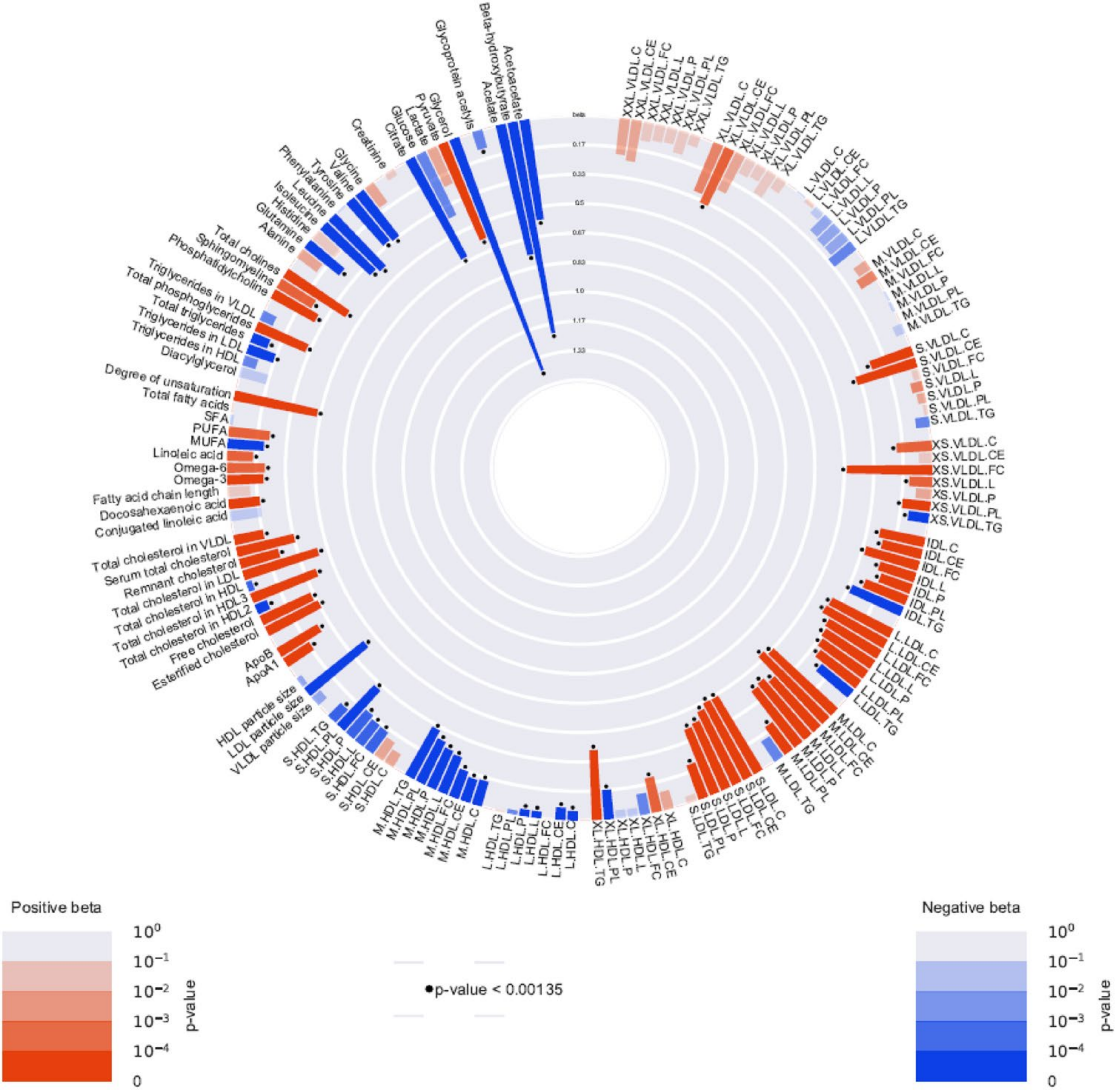
Circular plot of metabolomic measures after the low dose insulin infusion compared with baseline. Red bars stand for positive betas and blue bars stand for negative betas. The floating dots represents the significance of betas with standard of P-value < 0.00134

The standardized mean differences in metabolomic measures between baseline and low-dose insulin are summarized in Fig. 2, and presented in more detail in Supplementary Table 2. A total of 90 out of the 151 analyzed metabolomic measures significantly changed in concentration after 10 mM insulin infusion. In particular, after infusing low dose insulin for two hours, the concentrations of glycerol and three-hydroxybutyrate were materially decreased with betas of, respectively, − 1.41 [− 1.54, − 1.27] s.d. (P = 1.28e^−95^), and − 1.22 [− 1.36, − 1.07] s.d. (P = 1.44e^−61^). Other metabolomic measures that majorly decreased in concentration during this phase of the experiment included acetate (beta =  − 0.76 [− 0.88, − 0.63]; P = 1.52e^−32^), citrate (beta =  − 0.64 [− 0.82, − 0.46] s.d.; P = 6.95e^−12^), acetoacetate (beta =  − 0.57 [− 0.71, − 0.43] s.d.; P = 4.68e^−15^), LDL diameter (beta =  − 0.43 [− 0.56, − 0.30] s.d.; P = 2.71e^−11^) and medium-sized HDL. In contrast, the concentration of pyruvate, and the degree of fatty acid unsaturation increased with betas of 0.59 [0.40, 0.78] s.d. (P = 7.46e^−10^) and 0.48 [0.38, 0.57] s.d (P = 1.53e^−22^) respectively. In addition, the majority of LDL-sized particles and the concentration of apo-lipoprotein B (ApoB) increased in concentration after low-dose insulin infusion.

### Changes in metabolomic measures at high-dose insulin infusion

**Fig. 3:**
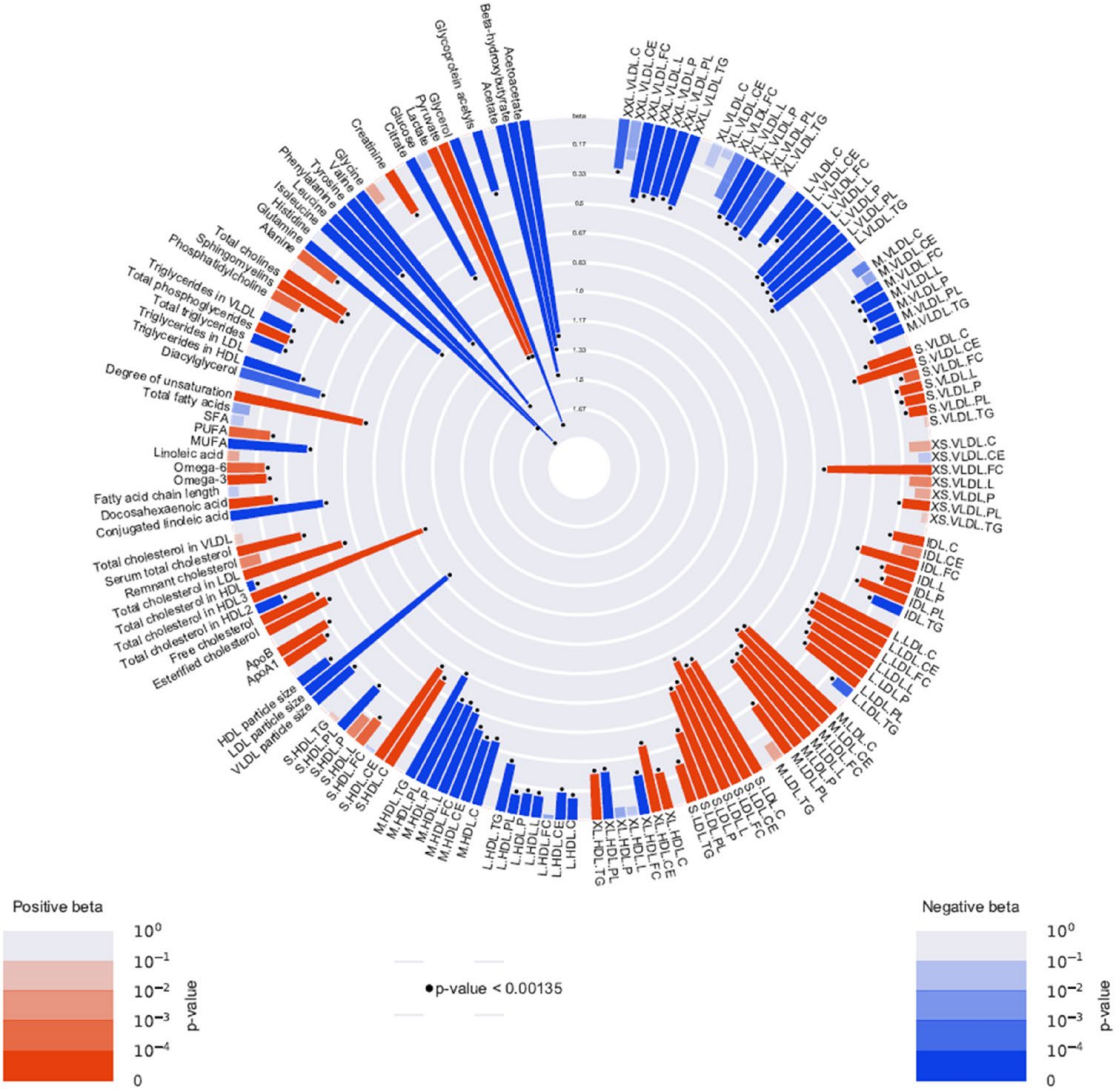
Circular plot of metabolomic measures after high dose insulin infusion compared with baseline. Red bars stand for positive betas and blue bars stand for negative betas. The floating dots represents the significance of betas with standard of P-value < 0.00134

Figure 3 summarizes the mean changes of metabolomic measures after high dose insulin infusion, and Supplementary Table 3 presents the results in more detail. 121 out of the 151 metabolomic measures changed significantly in concentration with 40 mM insulin infusion compared with the baseline. The concentrations of glycerol, leucine, isoleucine and valine largely decreased with betas of, respectively, − 1.72 [− 1.85, − 1.59] s.d. (P = 6e^−142^), − 1.78 [− 1.88, − 1.69] s.d. (P = 2.7e^−295^), − 1.65 [− 1.77, − 1.54] s.d. (P = 3.8e^−174^) and -1.53 [− 1.63, − 1.44] s.d. (P = 6.6e^−243^) compared with baseline. In addition, the concentrations of acetate, three-hydroxybutyrate, acetoacetate, tyrosine, glutamine and citrate also decreased. The concentrations of medium, large, extra-large and super extra-large VLDL particles and medium HDL and large HDL decreased after the high-dose insulin infusion. The concentration of pyruvate, lactate, total cholesterol in HDL3, and the degree of fatty acid unsaturation increased significantly with betas of, respectively, 1.29[1.10, 1.48] s.d. (P = 2.15e^−41^), 1.29 [1.09, 1.49] s.d. (P = 1.91e^−37^), 1.04 [0.91, 1.17] s.d. (P = 1.40e^−54^) and 0.74 [0.65, 0.84] s.d. (P = 2.40e^−51^). The concentrations of almost all LDL-sized particles, small and extra small VLDL and ApoB also increased significantly after the high dose insulin infusion. In addition, LDL diameter decreased.

**Fig. 4:**
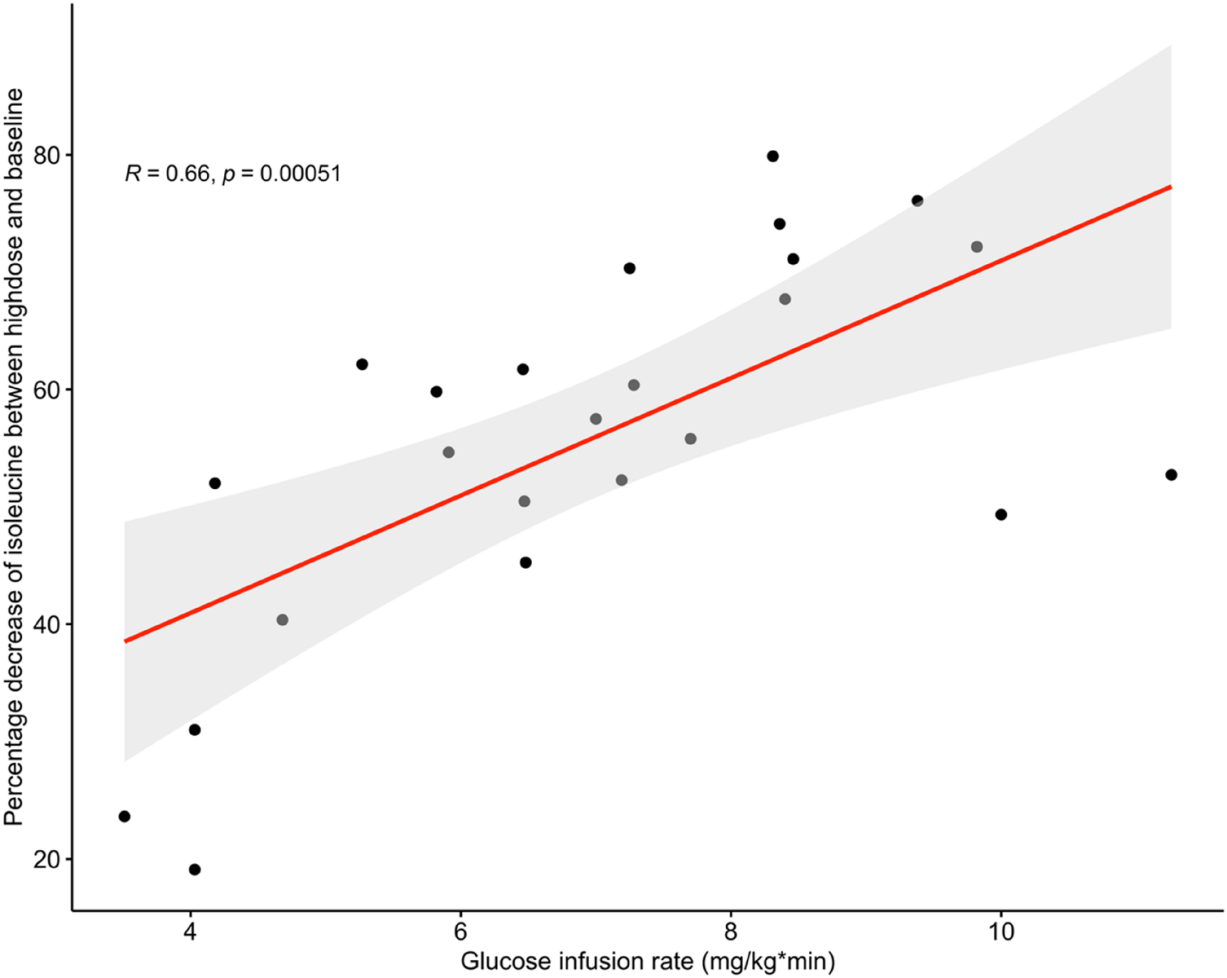
Percentage changes of isoleucine in high dose insulin infusion compared with baseline. Black points represent individuals. Red line is regression line and light grey area represent 95% confidence interval

Figure 4 showed the percentage changes of isoleucine between high dosage insulin and baseline, which indicated that the magnitude of the changes in isoleucine was correlated with glucose infusion rate. A stronger decrease of isoleucine concentration was found in individuals with higher glucose infusion rates. Similar patterns of change in leucine and valine dependent on the glucose infusion rates were observed (Supplementary Figs. 2 and 3). Changes in BCAAs at high dose insulin infusion were also positively correlated with glucose disposal rate (GDR) with r = 0.68, p = 0.00022, r = 0.61, p = 0.0017, and r = 0.5, p = 0.012, for isoleucine, leucine, and valine respectively.

### Differential changes in metabolomic measures between high and low dose insulin infusion

**Fig. 5:**
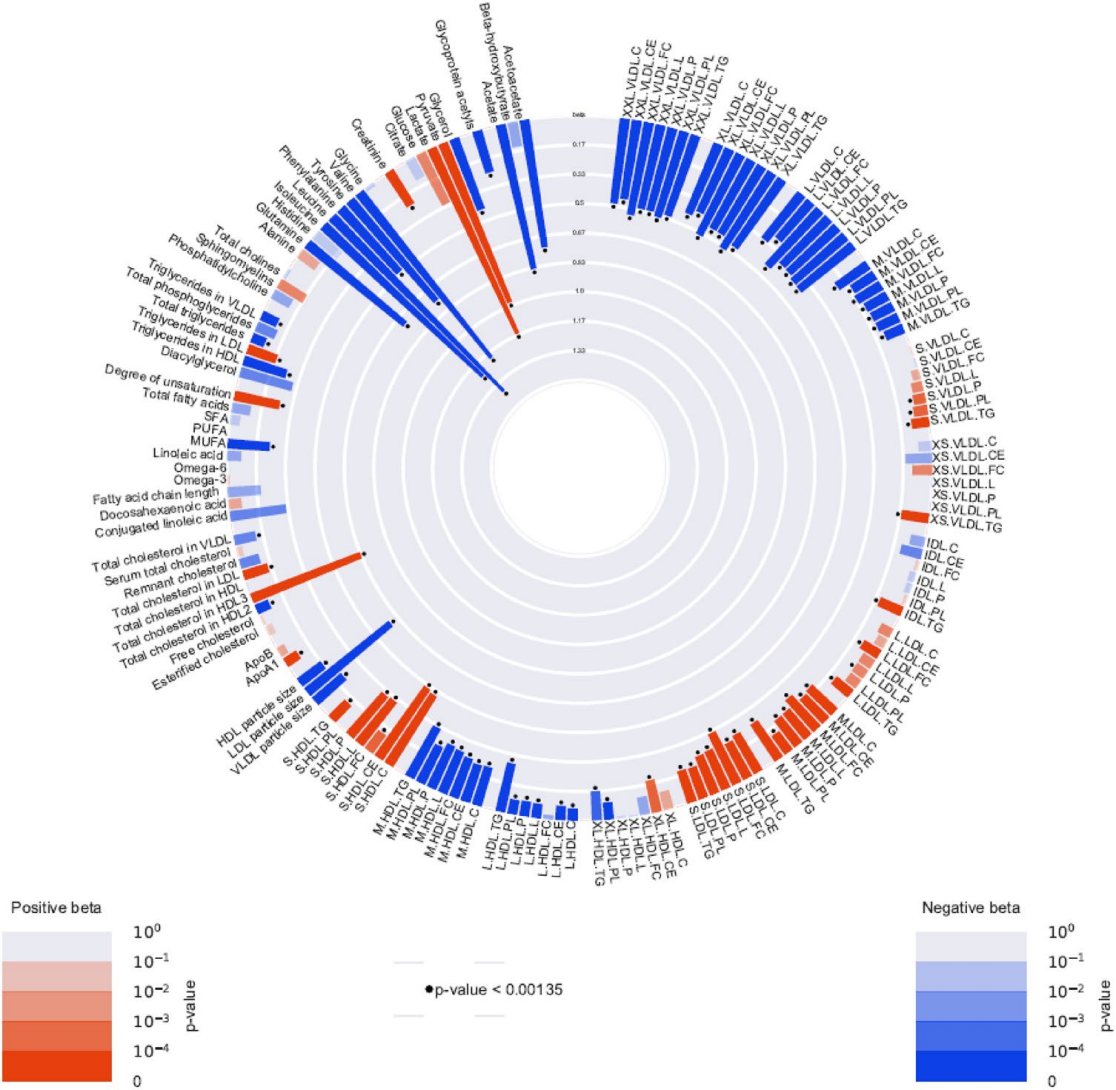
Circular plot of changes in metabolomic measures after high dose insulin infusion compared with low dose insulin infusion. Red bars stand for positive betas and blue bars stand for negative betas. The floating dots represents the significance of betas with standard of P-value < 0.00134

Figure 5 shows that 99 metabolomic measures changed significantly after high dose insulin infusion compared with low dose insulin infusion and Supplementary Table 4 provides the results in detail. Apparent additional decreases were specifically seen in the concentrations of branched-chain amino acids, acetate, tyrosine, acetoacetate, glutamine and LDL diameter. Among these significantly changed metabolomic measures, the largest additional changes of concentrations were in branched-chain amino acids, which decreased with betas of − 1.39 [− 1.47, − 1.30] s.d. (P = 2.4e^−244^) for leucine, − 1.24 [− 1.35, − 1.14] s.d. (P = 1.6e^−123^) for isoleucine and − 1.20 [− 1.27, − 1.12] s.d. (P = 5.8e^−211^) for valine compared with low-dose insulin infusion. Furthermore, the concentrations of acetate, tyrosine, acetoacetate, glutamine, extra-large and super extra-large VLDL particles also sharply decreased. In contrast, the concentrations of lactate (beta = 1.16 [0.97, 1.35] s.d.; P = 2.38e^−33^), pyruvate (beta = 0.98 [0.80, 1.16] s.d.; P = 1.95e^−27^) and HDL3C (beta = 0.67[0.54, 0.79] s.d.; P = 3.72e^−25^) greatly increased. In addition, LDL diameter further decreased.

**Fig. 6:**
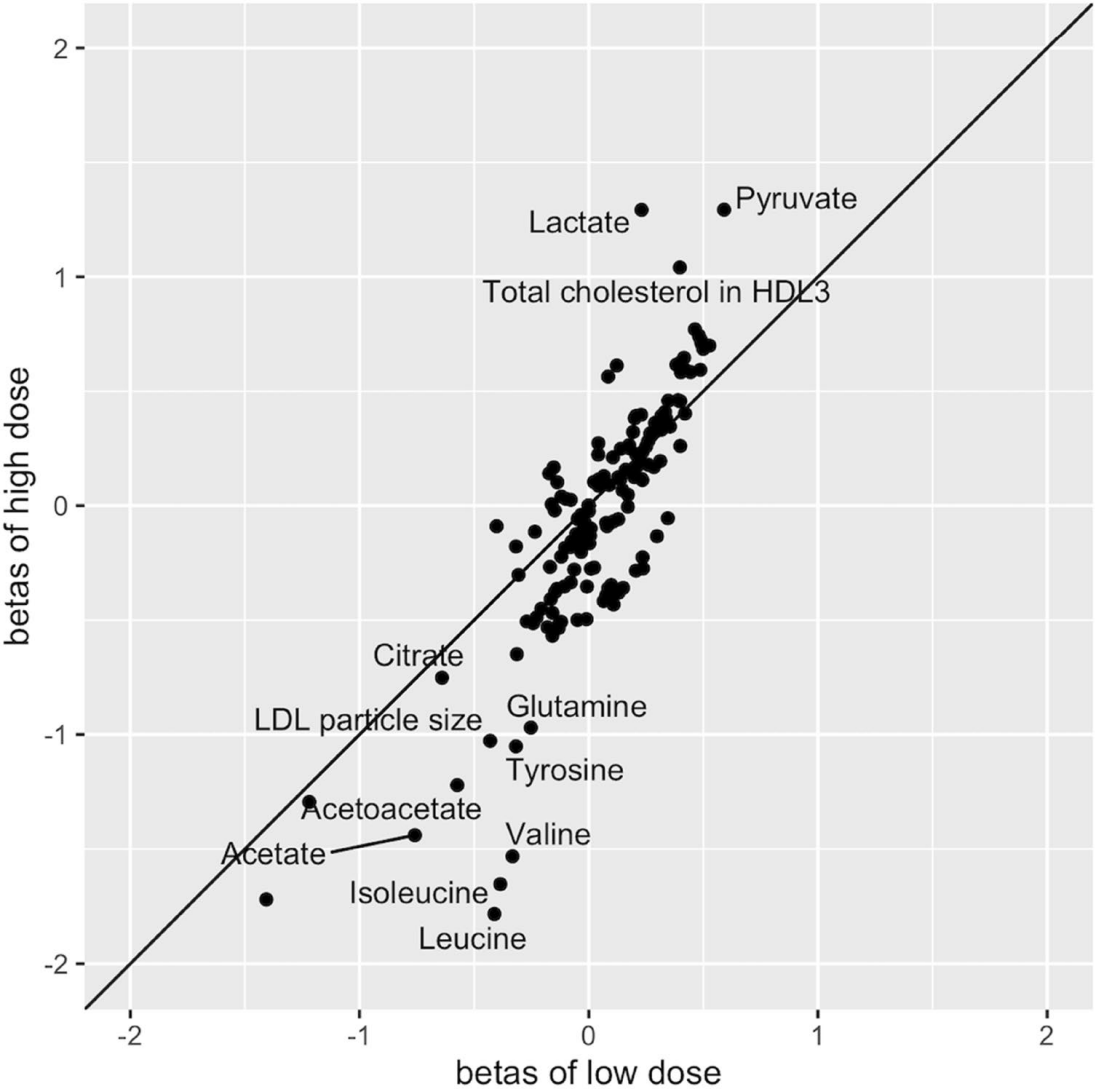
Beta-beta plot of high dose insulin infusion versus low dose insulin infusion. Metabolomic measures on the diagonal line have reached their maximal response already at low dose insulin infusion, whereas those that deviate from this line show a dose-dependent response

Based on the results from the low- and high-dose analyses, a beta-beta plot comparing low-dose insulin infusion and high-dose insulin infusion was generated (Fig. 6). Metabolomic measures on the diagonal line (Y = X) have reached their maximal response already at low-dose insulin infusion, whereas those that deviate from this line show a dose-dependent response. Most notably, leucine, isoleucine, valine and lactate, but also LDL diameter showed a clear additional effect at high-dose insulin infusion beyond that of low-dose insulin infusion.

## Conclusions and discussion

This study explored the changes of blood metabolomic measures in 24 clinically healthy individuals during a hyperinsulinemic euglycemic clamp study. We found that a large number of metabolomic measures changed significantly in concentration in response to low- and high-dose insulin infusion under euglycemic conditions. A total of 90 out of the 151 analyzed metabolomic measures changed significantly at low-dose insulin infusion, while 121 metabolomic measures changed significantly at high-dose insulin infusion. Some metabolomic measures seemed to have reached their maximum response already at low-dose (10 mU/m^2^/min) insulin infusion, whereas other metabolomic measures showed an additional response at high-dose (40 mU/m^2^/min)) insulin infusion. This shows that ^1^H-NMR-based metabolomic measures are differentially insulin sensitive.

At low-dose insulin infusion, the largest changes in metabolomic measures comprised glycerol, pyruvate, citrate, three-hydroxybutyrate, acetate, acetoacetate and LDL diameter. These changes are thought to mainly occur via the liver. Low-dose hyperinsulinemia-euglycemia stimulates glycolysis in the liver. This results in the increased production and turnover of pyruvate, which could leak into the circulation (Guo, 2012). Glycolysis also increases the demand on the mitochondrial citric acid cycle, which requires citrate and this could explain the decrease in citrate. A potential alternative explanation for the observation of the decrease in glycerol after low-dose (10 mU/m^2^/min) insulin infusion is an effect of insulin on inhibiting hormone sensitive lipase which would cause a decrease in adipose tissue lipolysis. It had been shown previously that suppression of lipolysis in adipose tissue is very insulin sensitive, and differences in adipose tissue lipolysis between individuals with type 1 diabetes and healthy controls could be detected at insulin doses as low as 4 mU/m^2^/min (Schauer, 2011). It is a limitation of the current study that, although (tracer) data on the rate of glycerol appearance were available, the insulin dosages applied might not be low enough to accurately assess potential differences in suppression of lipolysis in adipose tissue (and relate these to some of the observed changes in metabolites). After an overnight fast, ketogenesis is activated in the liver to meet the energy demand of the body (in particular the brain) and ketone bodies such as three-hydroxybutyrate, acetate and acetoacetate are formed in this process (Barnett & Barnett, 2003). After the infusion of glucose and insulin during the clamp procedure, which is performed in the fasted state, the secretion of ketone bodies is acutely inhibited which explains their decreased concentrations (Ciaraldi, 2004).

At high-dose intravenous insulin infusion, the largest changes in metabolomic measures included not only glycerol, pyruvate, lactate, citrate, three-hydroxybutyrate, acetate, acetoacetate, but also leucine, isoleucine, valine, tyrosine, and glutamine. In addition, all sizes of VLDL particles decreased, all sizes of LDL particles increased and mean LDL diameter decreased. High-dose insulin infusion is thought to affect processes in peripheral tissues such as muscle and fat in addition to processes in the liver. In muscle, insulin promotes the synthesis of proteins and suppresses proteolysis (Lukens, 1964), which could explain the observed large decrease in concentrations of amino acids including leucine, isoleucine, valine, tyrosine and glutamine. The observed correlation between percentage changes of BCAAs and glucose infusion rate provides additional evidence that the observed decrease of BCAAs at high dose-insulin infusion are positively related to whole-body insulin sensitivity. In addition to these effects, the decrease of medium, large, extra-large and super extra-large VLDL particles, and the increase of the majority of LDL particles, small VLDL, extra small VLDL and cholesterol concentration are indicative of an increased clearance of triglycerides. Triacylglycerol in the large VLDL particles is hydrolyzed by lipoprotein lipase resulting in the formation of smaller VLDL and LDL (Bhagavan & Ha, 2015).

The increased concentration of ApoB at low-dose insulin infusion could be explained by stimulation of de-novo lipogenesis in the liver and an increased VLDL production (Julius, 2003). Interestingly, high-dose insulin infusion had a more pronounced effect on the lipoprotein profile without additionally affecting the concentration of ApoB. This could be explained by direct or indirect high-dose insulin induced changes in the activity of plasma proteins that affect the interchange of components between lipoproteins in the plasma, such as phospholipid transfer protein (PLTP) and cholesteryl ester transfer protein (CETP) (Feingold & Grunfeld, 2000; Van Tol et al., 1997).

Multiple of the metabolomic measures that we identify as being insulin dependent in the present study have been described before in relation to cardiovascular disease risk. For example, triglycerides within all lipoproteins have been associated with increased risk of incident myocardial infraction and ischemic stroke (Holmes, 2018). In addition, high cholesterol levels in HDL particles have been associated with a lower risk of developing coronary heart disease, myocardial infraction and ischemic stroke (Joshi, 2020). A main driver of atherosclerotic cardiovascular disease is LDL cholesterol which is increased by low-dose insulin and further increased by high-dose insulin infusion (Ference, 2017). Additionally, the increase of LDL particle number and the decrease of LDL particle size have also been associated with increased risk of cardiovascular disease (Campos, 1992). Both parameters are found to be insulin dose dependent in our study. Moreover, the increase of beta-hydroxybutyrate in circulating blood has been associated with an increased intracranial carotid artery atherosclerosis (Vojinovic, 2018). Branched-chain amino acid were also identified to be associated with incident cardiovascular disease (Tobias Deirdre, 2018). The insulin dose dependently increased levels of these cardiovascular-disease risk associated metabolomic measures suggest that increased insulin has atherogenic properties independent of glucose concentrations.

Some metabolomic measures identified to be insulin sensitive in our study have also been identified as potential biomarkers for the risk to develop type 2 diabetes. Specifically, higher levels of the BCAAs (leucine, isoleucine and valine) and the aromatic amino acids (tyrosine and phenylalanine) have been associated with increased risk of type 2 diabetes and have the potential to predict the future development of diabetes (Wang, 2011). These amino acids were also among the metabolites that showed the largest changes in response to the high dose insulin infusion in our study. These data could be interpreted as indicating that decreased insulin sensitivity of amino acids leucine, isoleucine, valine, tyrosine and phenylalanine are predictive for the increased risk of developing type 2 diabetes. However, it has also been demonstrated by Mendelian randomization analysis that higher levels of the branched chain amino acids themselves are causally associated with the risk of type 2 diabetes (Lotta, 2016). Whether increased levels of branched chain amino acids are both consequence and cause of insulin resistance/type 2 diabetes remains to be established.

Our study has provided insight into the direct effects of insulin on changes of metabolomic measures in apparently healthy people under euglycemic conditions. A limitation of this study is the limited sample size, which does not allow specific subgroup analyses. In addition, the age of participants ranged from 50 to 75 years old, which means the results might not apply to younger ages. Moreover, it is important to note that the present study population was selected based on their health and partly on their propensity to become long-lived. This might have introduced bias in our study.

In conclusion, the majority of the plasma metabolomic measures determined by an 1H-NMR metabolomics platform are sensitive to insulin and a large fraction of these responses are insulin dose-dependent. It thus seems likely that some of these metabolomics measures will be differentially affected by the development of insulin resistance. Since low- and high-dose insulin levels are assumed to target, respectively, the liver and the liver plus peripheral organs (i.e. muscle and fat), our data provide insight into the direct role of insulin on specific processes in the liver and the peripheral tissues. Moreover, our data showed insulin-specific effects on metabolomic measures such as LDL particle number and size, which have previously associated with an increased risk of cardiovascular disease. The implications of this study are to not only avoid the chronic hyperinsulinemia that is associated with insulin resistance, but also to avoid frequent hyperinsulinemia that is caused by frequent snacking as a means to reduce exposure to an atherogenic lipoprotein profile.

## Supplementary Information

Below is the link to the electronic supplementary material.

Supplementary file1 (PDF 340 kb)

Supplementary file2 (XLSX 74 kb)

## Data Availability

Due to ethical constraints, data from the study is not freely available. Data is available on request after approval of a research proposal by the board of the Leiden Longevity Study.

## Code availability

The present study uses open source software and statistical packages. No in-house made codes were used.

## References

[CR1] American Diabetes A (2010). Diagnosis and classification of diabetes mellitus. Diabetes Care.

[CR2] Barnett CR, Barnett YA, Caballero B (2003). Ketone bodies. Encyclopedia of food sciences and nutrition.

[CR3] Bazotte RB, Silva LG, Schiavon FP (2014). Insulin resistance in the liver: Deficiency or excess of insulin?. Cell Cycle (georgetown, Tex).

[CR4] Beger RD (2016). Metabolomics enables precision medicine: "A White Paper, Community Perspective". Metabolomics.

[CR5] Bhagavan NV, Ha C-E, Bhagavan NV, Ha C-E (2015). Chapter 18 - Lipids III: Plasma lipoproteins. Essentials of medical biochemistry.

[CR6] Bukowiecka-Matusiak M, Chmielewska-Kassassir M, Szczesna D, Wozniak LA (2016). Metabolomic insight into lipid and protein profile in diabetes using mass spectrometry. Mini Reviews in Medicinal Chemistry..

[CR7] Campos H (1992). Low density lipoprotein particle size and coronary artery disease. Arteriosclerosis and Thrombosis: A Journal of Vascular Biology.

[CR8] Ciaraldi TP, Henry RR (2004). Insulin regulation of ketone body metabolism international textbook of diabetes mellitus.

[CR9] Dimitriadis G, Mitrou P, Lambadiari V, Maratou E, Raptis SA (2011). Insulin effects in muscle and adipose tissue. Diabetes Research and Clinical Practice.

[CR10] Feingold KR, Grunfeld C (2000). Introduction to lipids and lipoproteins.

[CR11] Ference BA (2017). Low-density lipoproteins cause atherosclerotic cardiovascular disease. 1. Evidence from genetic, epidemiologic, and clinical studies. A consensus statement from the European Atherosclerosis Society Consensus Panel. European Heart Journal.

[CR12] Finegood DT, Bergman RN, Vranic M (1987). Estimation of endogenous glucose production during hyperinsulinemic-euglycemic glucose clamps. Comparison of unlabeled and labeled exogenous glucose infusates. Diabetes.

[CR13] Guo X (2012). Glycolysis in the control of blood glucose homeostasis. Acta Pharmaceutica Sinica B.

[CR14] Gutch M, Kumar S, Razi SM, Gupta KK, Gupta A (2015). Assessment of insulin sensitivity/resistance. Indian Journal of Endocrinology and Metabolism.

[CR15] Holmes MV (2018). Lipids, lipoproteins, and metabolites and risk of myocardial infarction and stroke. Journal of the American College of Cardiology.

[CR16] Joshi R (2020). Triglyceride-containing lipoprotein sub-fractions and risk of coronary heart disease and stroke: A prospective analysis in 11,560 adults. European Journal of Preventive Cardiology.

[CR17] Julius U (2003). Influence of plasma free fatty acids on lipoprotein synthesis and diabetic dyslipidemia. Experimental and Clinical Endocrinology & Diabetes.

[CR18] Kettunen J (2016). Genome-wide study for circulating metabolites identifies 62 loci and reveals novel systemic effects of LPA. Nature Communications.

[CR19] Knebel B (2016). Specific metabolic profiles and their relationship to insulin resistance in recent-onset type 1 and type 2 diabetes. The Journal of Clinical Endocrinology & Metabolism.

[CR20] Li J, Ji L (2005). Adjusting multiple testing in multilocus analyses using the eigenvalues of a correlation matrix. Heredity.

[CR21] Li J, Ji L (2005). Adjusting multiple testing in multilocus analyses using the eigenvalues of a correlation matrix. Heredity (edinb).

[CR22] Liu X, Locasale JW (2017). Metabolomics: A primer. Trends in Biochemical Sciences.

[CR23] Lotta LA (2016). Genetic predisposition to an impaired metabolism of the branched-chain amino acids and risk of type 2 diabetes: A mendelian randomisation analysis. PLOS Medicine.

[CR24] Lukens FDW (1964). Insulin and protein metabolism. Diabetes.

[CR25] Muniyappa R, Lee S, Chen H, Quon MJ (2008). Current approaches for assessing insulin sensitivity and resistance in vivo: Advantages, limitations, and appropriate usage. American Journal of Physiology-Endocrinology and Metabolism.

[CR26] Ormazabal V, Nair S, Elfeky O, Aguayo C, Salomon C, Zuñiga FA (2018). Association between insulin resistance and the development of cardiovascular disease. Cardiovascular Diabetology.

[CR27] Phillips SM (2008). Insulin and muscle protein turnover in humans: Stimulatory, permissive, inhibitory, or all of the above?. American Journal of Physiology. Endocrinology and Metabolism.

[CR28] R Development Core Team. (2019). *R: A language and environment for statistical computing*. Vienna, Austria: R Foundation for Statistical Computing.

[CR29] Roberts CK, Hevener AL, Barnard RJ (2013). Metabolic syndrome and insulin resistance: Underlying causes and modification by exercise training. Comprehensive Physiology.

[CR30] Saccà L, Cicala M, Trimarco B, Ungaro B, Vigorito C (1982). Differential effects of insulin on splanchnic and peripheral glucose disposal after an intravenous glucose load in man. The Journal of Clinical Investigation.

[CR31] Schauer IE (2011). Insulin resistance, defective insulin-mediated fatty acid suppression, and coronary artery calcification in subjects with and without type 1 diabetes: The CACTI study. Diabetes.

[CR32] Schoenmaker M (2006). Evidence of genetic enrichment for exceptional survival using a family approach: The Leiden Longevity Study. European Journal of Human Genetics.

[CR33] Shaham O (2008). Metabolic profiling of the human response to a glucose challenge reveals distinct axes of insulin sensitivity. Molecular Systems Biology.

[CR34] Soininen P, Kangas AJ, Würtz P, Suna T, Ala-Korpela M (2015). Quantitative serum nuclear magnetic resonance metabolomics in cardiovascular epidemiology and genetics. Circulation.

[CR35] Steele R (1959). Influences of glucose loading and of injected insulin on hepatic glucose output. Annals of the New York Academy of Sciences.

[CR36] Taylor R (2012). Insulin resistance and type 2 diabetes. Diabetes.

[CR37] Tobias Deirdre K (2018). Circulating branched-chain amino acids and incident cardiovascular disease in a prospective cohort of US Women. Circulation.

[CR38] Tokarz VL, MacDonald PE, Klip A (2018). The cell biology of systemic insulin function. The Journal of Cell Biology.

[CR39] Van Tol A, Ligtenberg JJ, Riemens SC, van Haeften TW, Reitsma WD, Dullaart RP (1997). Lowering of plasma phospholipid transfer protein activity by acute hyperglycaemia-induced hyperinsulinaemia in healthy men. Scandinavian Journal of Clinical and Laboratory Investigation.

[CR40] Vojinovic D (2018). Metabolic profiling of intra- and extracranial carotid artery atherosclerosis. Atherosclerosis.

[CR41] Wallace TM, Levy JC, Matthews DR (2004). Use and abuse of HOMA modeling. Diabetes Care.

[CR42] Wang Q (2019). Insulin resistance and systemic metabolic changes in oral glucose tolerance test in 5340 individuals: An interventional study. BMC Medicine.

[CR43] Wang TJ (2011). Metabolite profiles and the risk of developing diabetes. Nature Medicine.

[CR44] Wijsman CA (2011). Familial longevity is marked by enhanced insulin sensitivity. Aging Cell.

[CR45] Wishart DS (2019). Metabolomics for investigating physiological and pathophysiological processes. Physiological Reviews.

[CR46] Yang Q, Vijayakumar A, Kahn BB (2018). Metabolites as regulators of insulin sensitivity and metabolism. Nature Reviews Molecular Cell Biology.

