# Supplementary material for: Differential insulin sensitivity of NMR-based metabolomic measures in a two-step hyperinsulinemic euglycemic clamp study
Source: Metabolomics. 2021 Jun 9;17(6):57. doi: 10.1007/s11306-021-01806-2 (PMC8190027; doi:10.1007/s11306-021-01806-2)
Supplement: Supplementary file 1 — Supplementary file1 (PDF 340 kb) [file 11306_2021_1806_MOESM1_ESM.pdf]

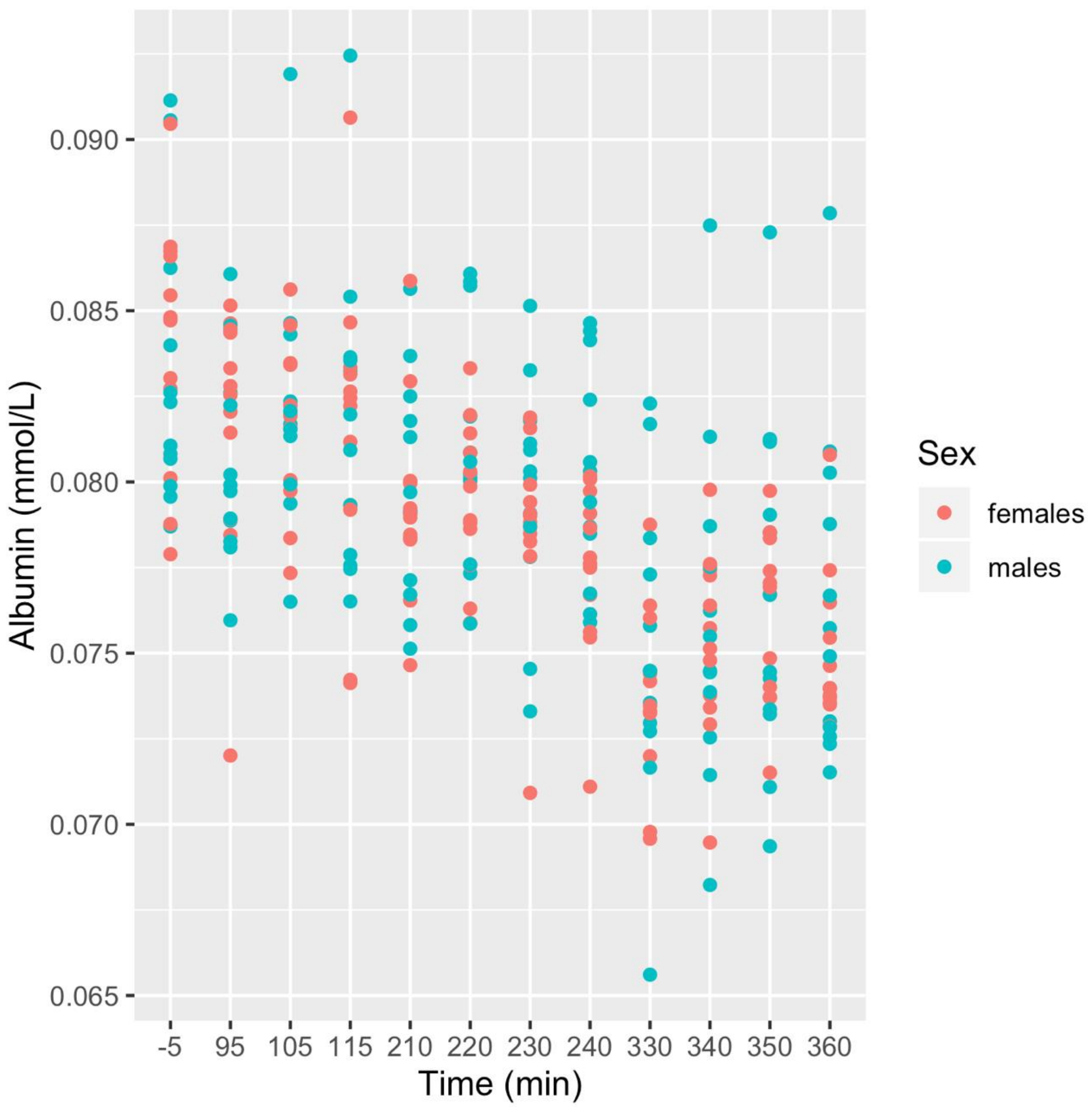

Supplementary Figure 1 Concentration of albumin over time. Blue points represent males and red points represent females.

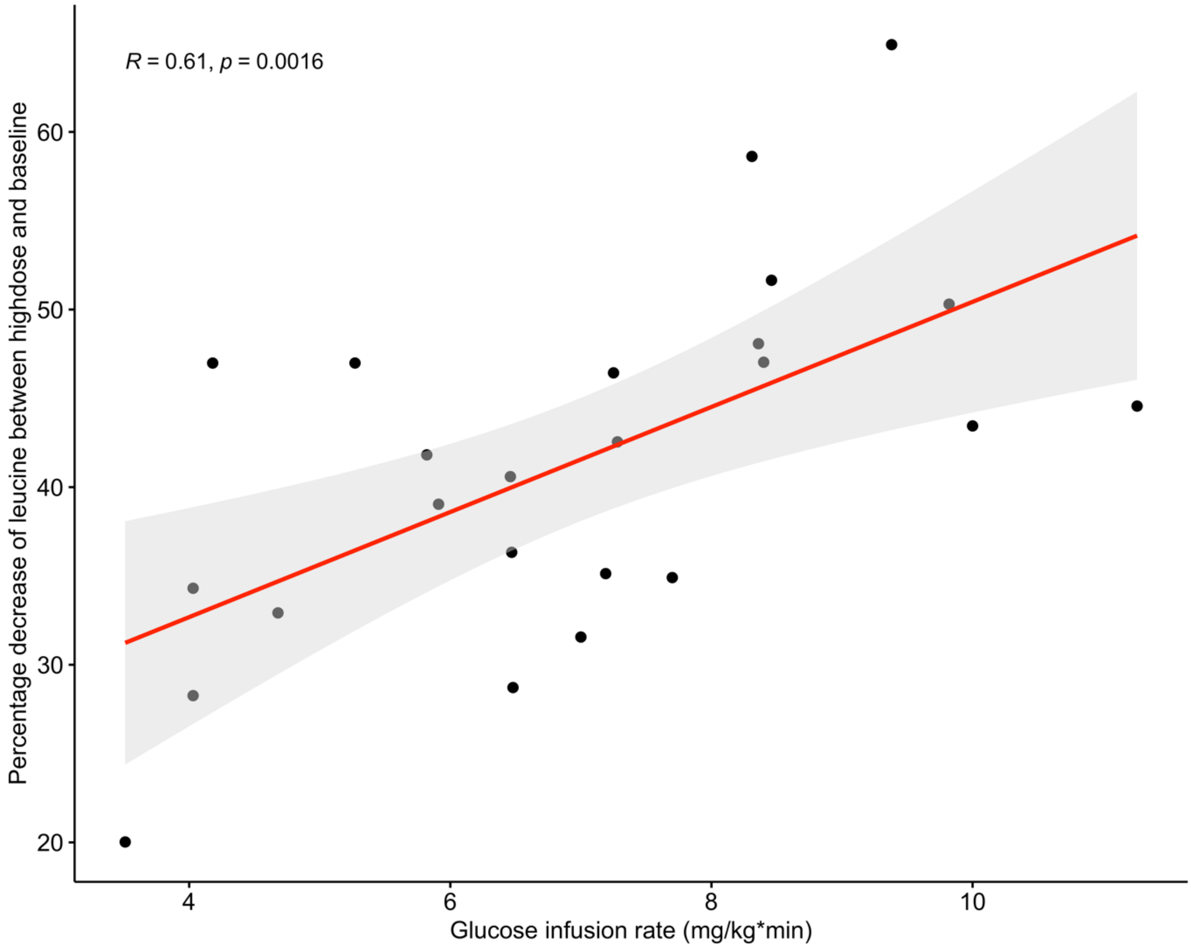

Supplementary Figure 2 Percentage decrease of leucine in high dose insulin infusion compared with baseline. Black points represent individuals. Red line is regression line and light grey area represent 95% confidence interval.

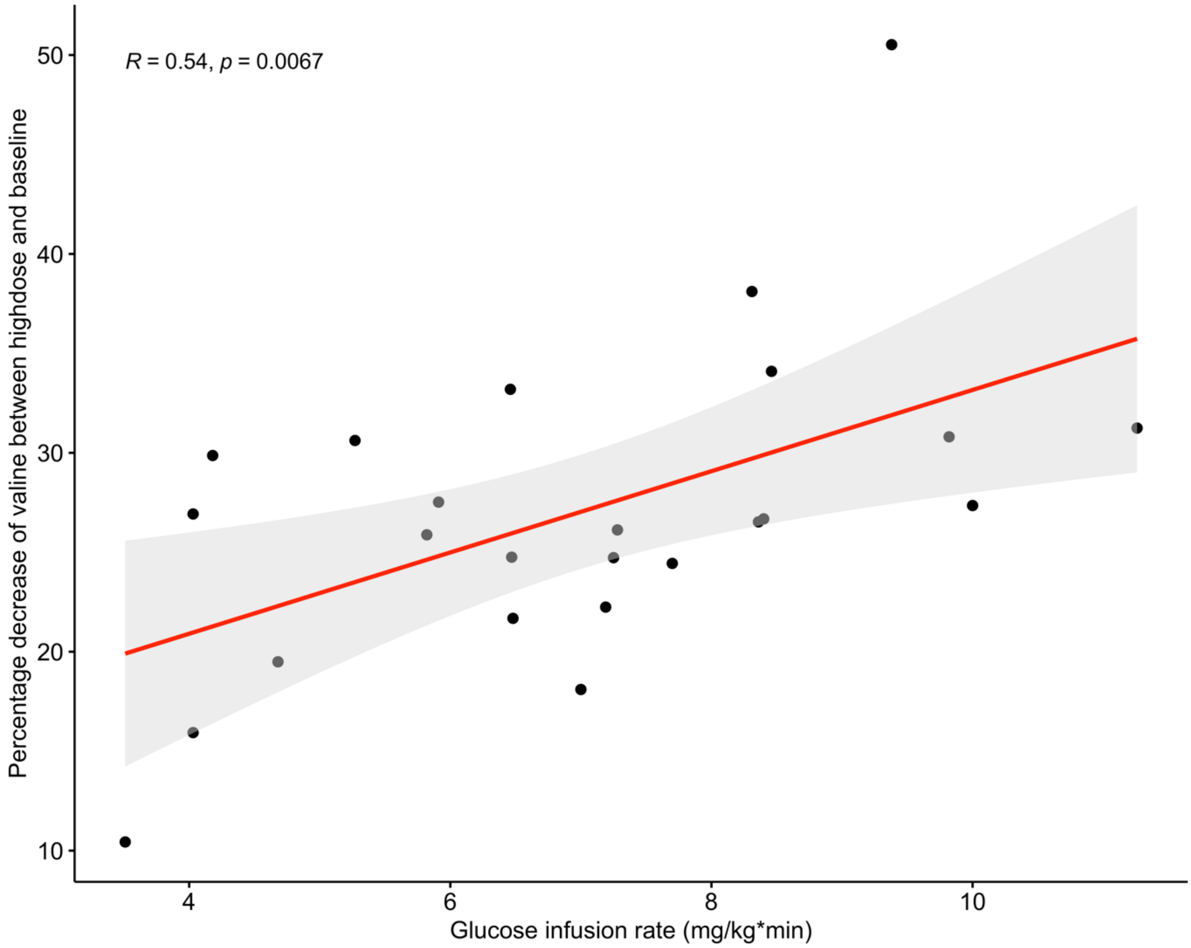

Supplementary Figure 3 Percentage decrease of valine in high dose insulin infusion compared with baseline. Black points represent individuals. Red line is regression line and light grey area represent 95% confidence interval.
